# A Missense Mutation in the Zinc Finger Domain of OsCESA7 Deleteriously Affects Cellulose Biosynthesis and Plant Growth in Rice

**DOI:** 10.1371/journal.pone.0153993

**Published:** 2016-04-19

**Authors:** Daofeng Wang, Yanling Qin, Jingjing Fang, Shoujiang Yuan, Lixiang Peng, Jinfeng Zhao, Xueyong Li

**Affiliations:** 1 National Key Facility for Crop Gene Resources and Genetic Improvement, Institute of Crop Science, Chinese Academy of Agricultural Sciences, Beijing, China; 2 Shandong Rice Research Institute, Jinan, China; Institute of Genetics and Developmental Biology, Chinese Academy of Sciences, CHINA

## Abstract

Rice is a model plant species for the study of cellulose biosynthesis. We isolated a mutant, S1-24, from ethyl methanesulfonate (EMS)-treated plants of the *japonica* rice cultivar, Nipponbare. The mutant exhibited brittle culms and other pleiotropic phenotypes such as dwarfism and partial sterility. The brittle culms resulted from reduced mechanical strength due to a defect in thickening of the sclerenchyma cell wall and reduced cellulose content in the culms of the S1-24 mutant. Map-based gene cloning and a complementation assay showed that phenotypes of the S1-24 mutant were caused by a recessive point mutation in the *OsCESA7* gene, which encodes cellulose synthase A subunit 7. The missense mutation changed the highly conserved C40 to Y in the zinc finger domain. The *OsCESA7* gene is expressed predominantly in the culm at the mature stage, particularly in mechanical tissues such as vascular bundles and sclerenchyma cells, consistent with the brittle phenotype in the culm. These results indicate that *OsCESA7* plays an important role in cellulose biosynthesis and plant growth.

## Introduction

Mechanical strength is an important agronomic trait in rice. It mainly affects lodging resistance and thus crop yield. Mechanical strength is provided primarily by the cell wall, and changes in either the structure or composition of the cell wall affects mechanical strength. Brittle culm (bc) mutants exhibit reduced mechanical strength in the culm and other organs. As a result, the culms of bc mutant plants can be easily broken. Bc-related mutants have been identified in several species including barley, rice, maize, and *Arabidopsis*.

The brittle phenotype is always directly related to the secondary cell wall. In higher plants, secondary cell wall is a fibrous system composed mainly of cellulose molecules [1]. Cellulose is the most important constituent of the secondary cell wall, which can reach 40–90% [2]. Changes in cellulose in secondary cell wall can always lead to the brittle culm phenotype. In addition, cellulose defects in secondary cell wall may also deleteriously affect morphology, fertility, and viability of the entire plant [3, 4].

Cellulose is a polymer of β-1,4-glucan, and its synthesis is generally catalyzed by a complex of various kinds of cellulose synthase (CESA) that use uridine diphosphate (UDP)-glucose as a substrate [5]. *CESA* is a large gene family with high conservation among most CESA proteins. Cellulose synthesis requires the cooperation of two or three different CESA subunits [2, 6].

At least 10 *CESA* (*AtCESA1–10*) genes have been found in *Arabidopsis* (http://www.arabidopsis.org). Studies on mutants showed that some of the *CESA* genes (*AtCESA4*, *AtCESA7*, and *AtCESA8*) were required for cellulose synthesis in the secondary cell wall [2, 3], while others (*AtCESA1*, *AtCESA2*, *AtCESA3*, *AtCESA5*, *AtCESA6*, and AtCESA9) were responsible for cellulose synthesis in the primary cell wall [4, 7]. All *CESA*-related mutants exhibited the brittle phenotype, diminished cellulose content, and decreased mechanical strength [8]. To date, several *AtCESA* genes and related mutants have been studied. The *irx3* mutant, which carries a mutation in the *AtCESA7* gene, exhibited not only decreased cellulose in culms but also a defect in the secondary walls in the xylem [9]. The same phenotype was also exhibited by the *irx1* and *irx5* mutants, which carry mutations in the *AtCESA3* and *AtCESA4* genes, respectively [10]. The *rsw1* mutant carrying a mutation in *AtCESA1* exhibited reduced cellulose and abnormal growth in elevated temperature [4]. The *prc1* mutant (*AtCESA6*) has the same phenotype as *rsw1*, and in addition, reduced cell length especially in roots and hypocotyledonary axes under darkness [7]. *Ixr1* (*AtCESA3)* and *ixr2 (AtCESA6)* were identified as insensitive mutants of cellulose synthesis inhibitor isoxaben [11, 12]. Furthermore, studies showed that the *AtCESA4*, *AtCESA7*, and *AtCESA8* genes were co-expressed in culms [13].

In rice, 11 *CESA* genes (*OsCESA1–11)* have been predicted by the Michigan State University (MSU) rice genome annotation project (http://rice.plantbiology.msu.edu/). Mutants of *OsCESA4*, *OsCESA7*, and *OsCESA9* were initially isolated by insertion of the endogenous retrotransposon *Tos17* [14]. These genes were orthologs of *AtCESAs* and involved mainly in secondary wall formation. Mutants of these genes exhibited defects in cellulose to different degrees and showed varied phenotypes. For example, two allelic mutants NC0259 and ND8759 of *OsCESA7* established by insertion of retrotransposon *Tos17* into the fifth exon and the seventh exon exhibited significantly reduced cellulose contents and abnormal plant growth (dwarfism, small leaves, withered leaf tips, and thin culms). Independent studies have shown that *bc7* and *bc11* are allelic mutants of *OsCESA4* [15, 16], and that *bc6* is a mutant of *OsCESA9* [17]. Cellulose content was reduced by 10% in the *bc7* mutant relative to the wild type. Sequence analysis revealed a 7-base deletion at the junction of exon 10 and intron 10 in the mutant that resulted in a reading frame shift and a consequent lack of functional protein [16]. In contrast, the cellulose content was reduced drastically in *bc11*. The site of the mutation in *bc11* is at a highly conserved residue located at the end of the fifth transmembrane domain. This point mutation decreased the abundance of OsCESA4 in the plasma membrane, probably due to a defect in the process of CESA complex secretion [15]. In the *bc6* mutant, which has a missense mutation in a highly conserved region of *OsCESA9*, the proportion of cellulose was reduced by 38%, while that of hemicellulose was increased by 34% [17]. Meanwhile, the amount of cellulose in the S1-60 mutant culms was reduced to 44.7% of that of wild-type plants by a recessive point mutation in *OsCESA9* (G905D) [18]. However, except the mutant established by insertion of the endogenous retrotransposon *Tos17*, there still no independent mutant of *OsCESA7* has been studied.

In this study, we isolated an ethyl methanesulfonate (EMS) induced rice mutant, S1-24, from the *japonica* cultivar Nipponbare. The mutant exhibited a brittle phenotype and abnormal growth. Our findings indicate that a missense mutation in *OsCESA7* leads to reduced cellulose levels and impaired cell wall thickness in the S1-24 mutant.

## Materials and Methods

### Plant materials and growth conditions

The S1-24 rice mutant exhibiting brittle culms was isolated from the *japonica* cultivar Nipponbare after EMS treatment. The brittle culm phenotype was stably inherited after continuous cultivation. An F_2_ mapping population was generated from a cross between S1-24 and Dular, a polymorphic *indica* cultivar. Rice plants were cultivated in an experimental field at the Shandong Rice Research Institute (Jining, Shandong, China) under natural growing conditions.

### Mechanical strength measurements

At the heading stage, the first and second internodes of wild-type and mutant plants were measured using an Instron 5848 MicoTester micro force/length testing device as reported previously [19]. The samples were cut into segments of equal length (8 cm) and used for measurement.

### Scanning electron microscopy

At the heading stage, fresh first-internode segments were excised from mutant and wild type plants and fixed in fixative solution (75% ethanol, 5% acetic acid, 5% glycerol, and 5% formaldehyde) for at least 1 day. Internode samples were then sectioned into thin pieces and dehydrated through an ethanol series. Specimens were critical point-dried, sputter-coated with gold, and observed with a scanning electron microscope (Quanta 200).

### Carbohydrate measurements

Fractionation and quantification of cell wall polysaccharides were carried out according to previously reported methods [20]. Briefly, at the heading stage, the second internodes of culms from mutant and wild-type plants were collected (0.5 g) and ground in liquid nitrogen to a fine powder, which was then washed with water. The samples were treated with 80% (v/v) ethanol at 100°C for 20 min and digested with 100 U of porcine pancreatic α-amylase in 50 mM MOPS–NaOH buffer (pH 6.5) at 37°C for 4 h. After removal of solubilized starch by centrifugation at 1500 g, the cell wall materials were sequentially extracted at 100°C for 10 min with hot water (hot water fraction), 50 mM EDTA (pH 6.8) (pectin fraction), and 17.5% (w/v) NaOH containing 0.04% NaBH_4_ (hemicellulose fraction). The hemicellulose fractions were then neutralized with acetic acid, separated at 4°C for 1 day, and dried. After washing with water, ethanol, and diethyl ether, the residual precipitate constituted the cellulose fraction. After extracting the four polysaccharide components, sugar content was assayed using a phenol–sulfuric acid method described previously [21].

### Mapping and sequencing

An F_2_ segregating population of plants generated from a cross between S1-24 and Dular was used for mapping and cloning of the target gene. For the primary mapping, a DNA pool composed of DNA from 10 individual mutant plants was used for bulked segregation analysis (BSA) [22]. Approximately 200 polymorphic indel markers distributed evenly throughout the rice genome were used for the BSA analysis. For fine mapping, new indel markers were designed by utilizing genomic sequence information for Nipponbare (http://rgp.dna.affrc.go.jp/) and Dular (our unpublished data). DNA was extracted from freshly frozen leaves of each plant. Polymerase chain reaction (PCR) was run using the following program: 5 min at 95°C followed by 35 cycles of 30 s at 94°C, 30 s at 55°C, 30 s at 72°C, and a final extension of 10 min at 72°C. PCR products were analyzed on a polyacrylamide gel stained with silver nitrate. The genomic region of the candidate gene was divided into several overlapping 1.5-kb fragments and amplified using high-fidelity PrimeSTAR DNA polymerase (TaKaRa). The PCR program parameters were 3 min at 94°C followed by 40 cycles at 98°C for 30 s, 55°C for 5 s, 72°C for 1.5 min, and a final extension at 72°C for 10 min. PCR products from the S1-24 mutant were sequenced directly.

### RT-PCR of *OsCESA7*

Total RNA was extracted from different tissues of wild-type rice plants. First-strand cDNA was synthesized using a Superscript III Reverse Transcription Kit (Invitrogen). Semiquantitative PCR was performed using LA Taq DNA polymerase (TaKaRa) with the rice *ACTIN1* gene serving as an internal control. The PCR program parameters were 1 min at 94°C followed by 30 cycles at 94°C for 30 s, 60°C for 30 s, 72°C for 30 s, and a final extension at 72°C for 10 min. Quantitative PCR was performed using a SYBR Premix Ex Taq2 kit (TaKaRa) under the following conditions: 10 s denaturing at 95°C, 30 s annealing at 60°C, 40 cycles, and run on an ABI PRISM 7900HT. The mRNA amount relative to ACTIN1 was calculated. Specific primers used for reverse transcription (RT)-PCR analysis were RT-F: 5’-TCCGTCGAGATCTTCATGAG-3’ and RT-R: 5’-ATGATGAACTTGCCGGTGAG-3’.

### GUS assay

β-Glucuronidase (GUS) activity was assayed according to a previous method [22]. An OsCESA7 promoter fragment (2,564 bp upstream of the start ATG) was amplified by PCR using specific primers: GUS-F, 5’-CTATGACATGATTACGAATTCGTCCAAGCAAGGTGACAGTA-3’ and GUS-R, 5’-GAAATTTACCCTCAGATCTACCATGGTGAGGTGCCGGGGAA-3’. The amplified promoter fragment was inserted into the binary vector pCAMBIA 1305.1 in frame with the *GUS* reporter gene. The construct was transformed into wild-type plants using an *Agrobacterium*-mediated transformation method [23]. Tissues from transgenic plants were stained for GUS activity in GUS staining solution (1 mM X-Gluc, 50 mM Na_2_HPO_4_–NaH_2_P_4_ (pH 7.2), 0.1% Triton X-100, and 20% methanol) for 24 h at 37°C. Tissues were observed after destaining in 70% ethanol.

### Complementation test

A 7,028-bp genomic DNA fragment containing the entire OsCESA7 coding region and the 2,760-bp upstream sequence was divided into two fragments and amplified by PCR using specific primers (24C1-F: 5’-TTGAATTCTGTGGGCACATGGAGGCGTA-3’, 24C1-R: 5’-TAATGGATCCACGACAGCGCGAACCACA-3’, 24C2-F: 5’-TATTGGATCCTCGACCAGCTGCCCAAGT-3’, and 24C2-R: 5’-GACACGTGTAATCTCCACCTCAGTTCTT-3’), respectively. The PCR program included 1 min at 94°C followed by 35 cycles at 94°C for 30 s, 60°C for 40 s, 72°C for 30 s, and a final extension at 72°C for 10 min. Then these two fragments were sequential inserted into the binary vector pCAMBIA1305.1 by special restriction sites (EcoRI & BamHI; BamHI & PmlI). The construct was transformed into S1-24 plants using an *Agrobacterium*-mediated transformation procedure [23].

### Sequence and phylogenetic analysis

Gene prediction was performed using online search software (http://rice.plantbiology.msu.edu/cgi-bin/gbrowse/rice/#). Exon/intron structures were identified by alignment of coding sequences (CDS) and genomic DNA sequences. Multiple sequence alignments were conducted using CLUSTALX software, and a phylogenetic tree was built using MEGA 4 software [24].

## Results

### The S1-24 mutant exhibits reduced mechanical strength

**Fig 1:**
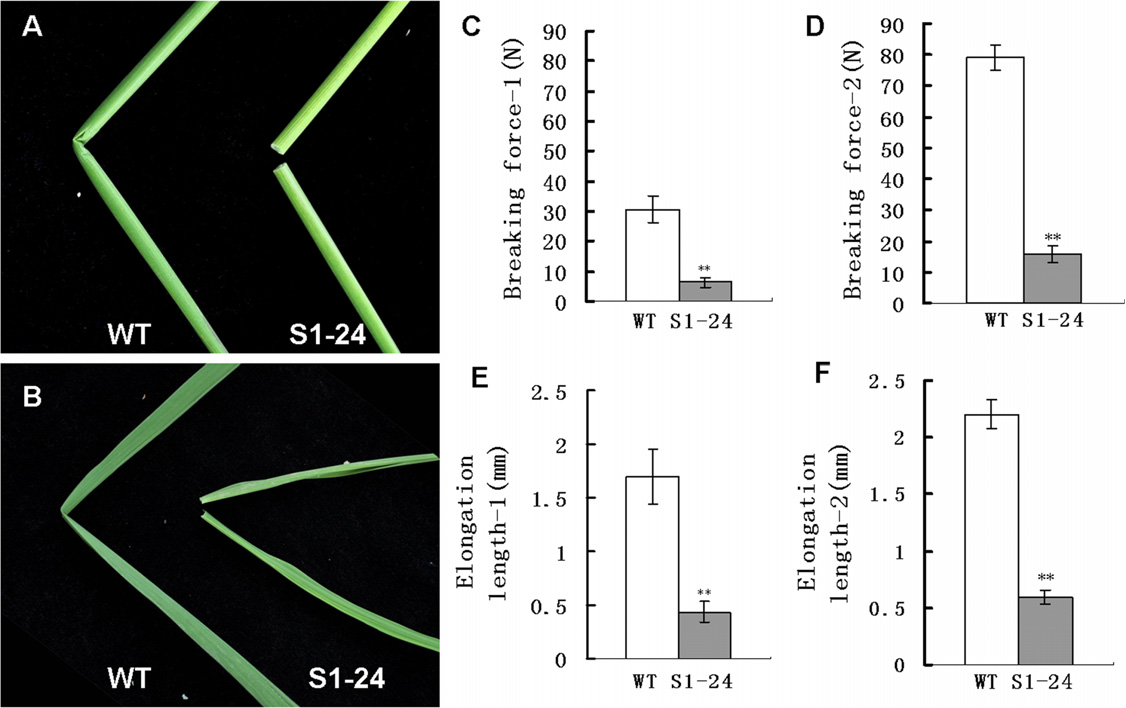
Phenotypes and physical properties of the S1-24 mutant. (A) An easily broken culm of S1-24 compared with the wild type. (B) An easily broken flag leaf of S1-24 compared with the wild type. (C, D) Force required to break the first and second upper internodes. (E, F) Elongation length of the first and second upper internodes. Values shown are the averages of values for five internodes. Bars represent standard errors. ** indicate statistical significance by a *t* test at *P* < 0.01.

The S1-24 mutant was isolated from plants of the *japonica* cultivar Nipponbare mutagenized with EMS; it is characterized by brittle culms and leaves that can be easily broken by bending (Fig 1A and 1B). Two parameters that are important for a precise description of this phenotype were measured: the breaking force and the elongation length, which are parameters that define the force required to break a culm segment and the elasticity of the plant tissue, respectively. We measured the mechanical strength of the first and second internodes of wild-type and mutant plants at the heading stage. The breaking force of the first and second upper internodes of the S1-24 mutant was reduced by 79% and 80%, respectively, compared with the wild-type plants (Fig 1C and 1D). As shown in Fig 1E and 1F, the elongation length of the first and second internodes of the mutant was reduced by 74% and 73%, respectively, compared with wild-type plants. These results showed that the mutation in S1-24 has a strong effect on the mechanical strength of rice plants.

**Fig 2:**
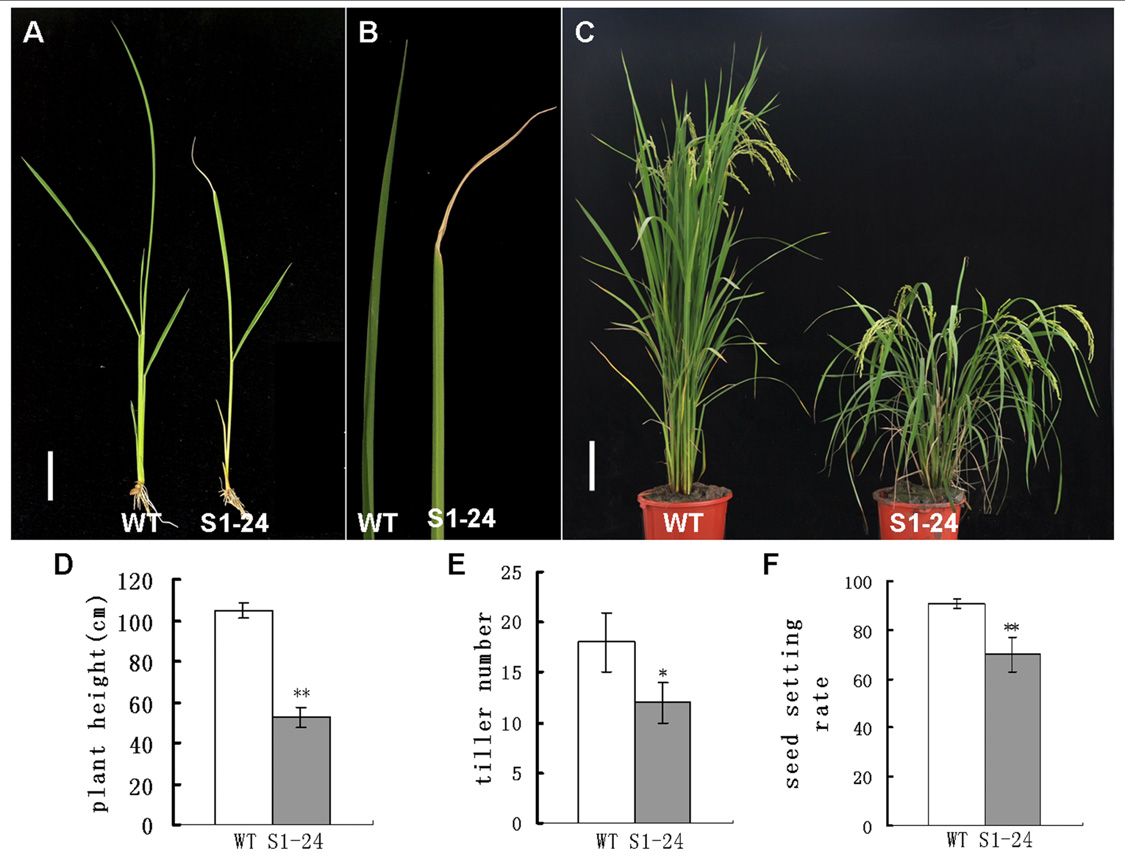
Pleiotropic phenotypes of the S1-24 mutant. (A) Gross morphology at the seedling stage (bar = 2 cm). (B) Withering in the flag leaf apex of the S1-24 mutant. (C) Gross morphology at the mature stage (bar = 12 cm). (D) Plant height at the mature stage. (E) Tiller number at the mature stage. (F) Seed setting rate. Values shown are the averages of values for 10 plants. Bars represent standard errors. ** indicates statistical significance by a *t* test at *P* < 0.01.

In addition to reduced mechanical strength, the S1-24 mutant exhibited other pleiotropic phenotypes. The mutant showed slight dwarfism with withered leaf tips at the seedling stage (Fig 2A and 2B). These phenotypes became more obvious at the mature stage. Leaves and stems with considerable drooping were observed (Fig 2C), and plant height of the S1-24 mutant was reduced to only 50% of that of wild-type plants (Fig 2D). In addition, the tiller number of the mutant was decreased by 33%, and the rate of seed setting decreased by approximately 23% (Fig 2E and 2F).

### Cell wall defect and change in cell wall composition

**Fig 3:**
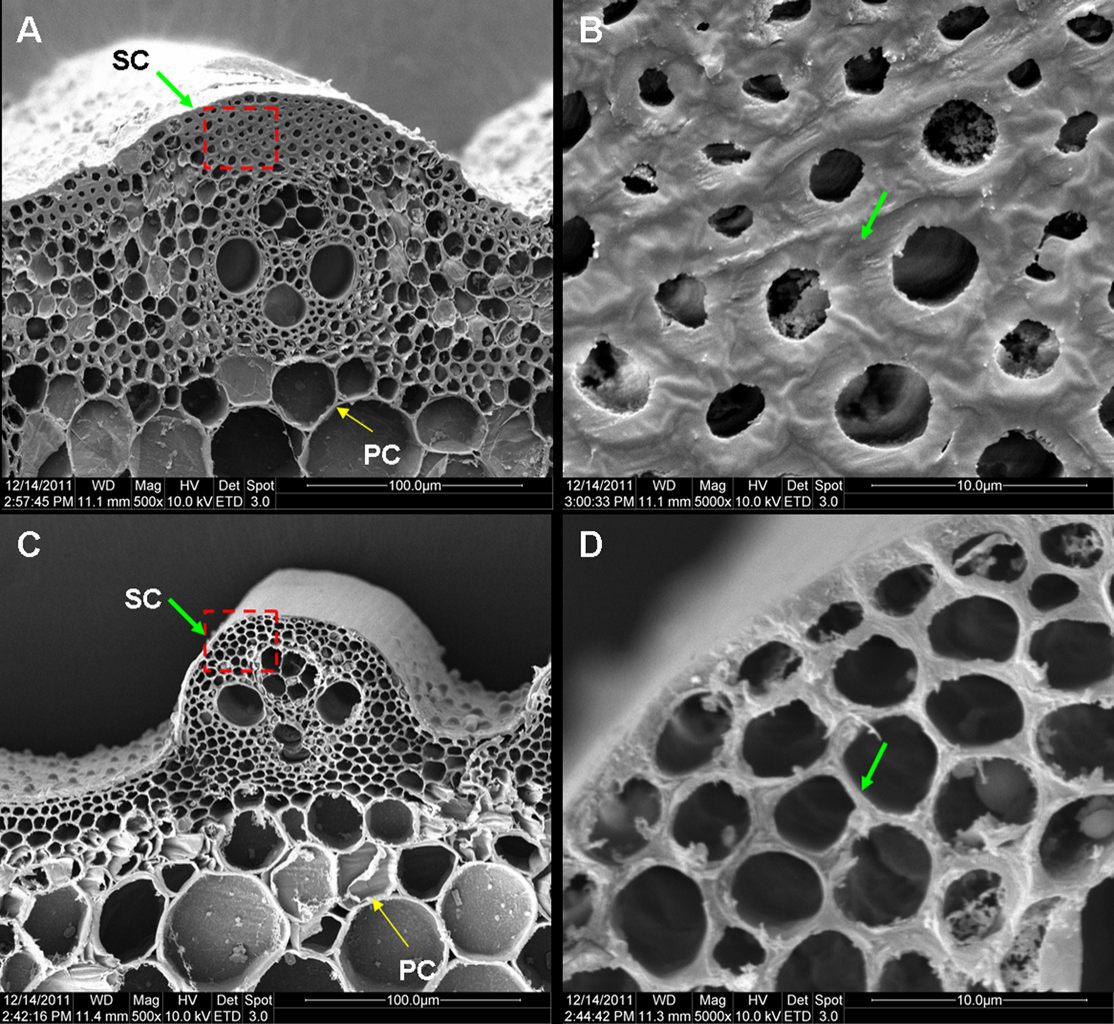
Cross section of a culm viewed under a scanning electron microscope. (A, B) Cross section of a wild-type culm. (C, D) Cross section of an S1-24 culm. Magnification in the images is 500× (A, C) or 5000 × (B, D). Green and yellow arrows represented thickened sclerenchyma cell (SC) walls and unthicken parenchyma cell (PC) walls.

To determine the cause of the reduction in mechanical strength in the S1-24 mutant, culm cross sections were examined under a scanning electron microscope. As shown in Fig 3, obvious differences were found between the mutant and wild-type culms. In the wild-type culms, the sclerenchyma cell walls, which provide the main structural support for the plant body, were obviously thick (Fig 3A and 3B). In contrast, the sclerenchyma cell walls in the mutant showed no significant thickening (Fig 3C and 3D). No obvious differences between parenchyma cells in wild-type and mutant culms were observed (Fig 3A and 3C). Consequently, the reduction in mechanical strength in the S1-24 mutant was due to a defect in thickening of the sclerenchyma cell wall.

**Fig 4:**
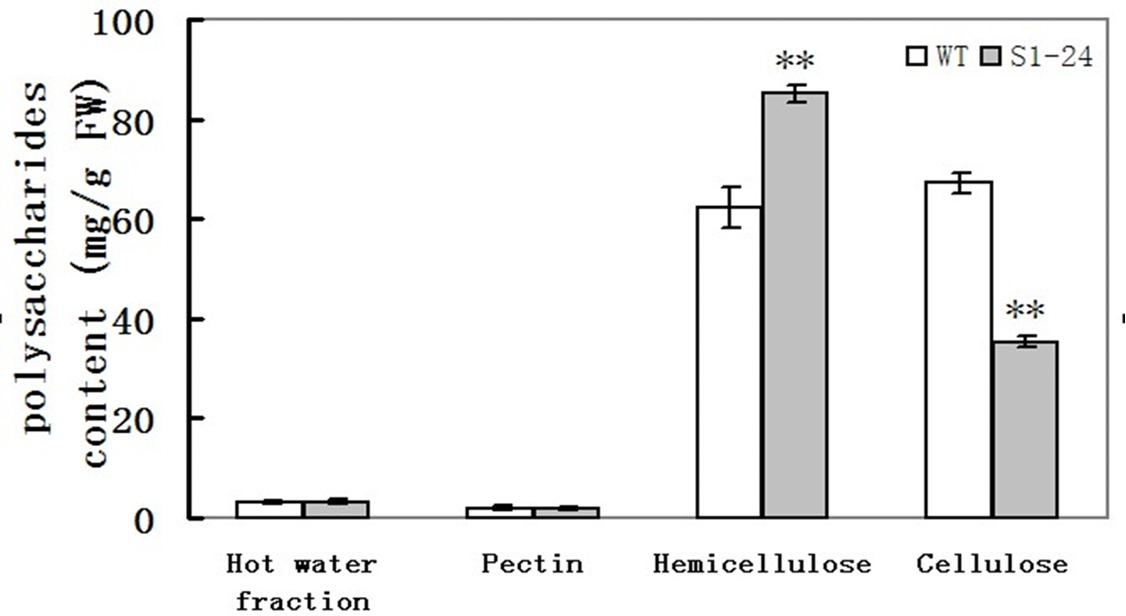
Polysaccharide content of cell walls in the second upper internodes at the heading stage. Values shown are averages of values for five plants. Bars represent standard errors. ** indicate statistical significance by a *t* test at *P* < 0.01.

The defect in cell wall thickening suggests that the cell wall composition might also be altered. To address this possibility, four polysaccharide fractions (hot water, pectin, hemicellulose, and cellulose), which are the main components of cell walls, were extracted from culms, and the proportions of each fraction were quantified. The amount of cellulose in S1-24 mutant culms was reduced to 48% that of wild-type culms (Fig 4). In contrast, the amount of hemicellulose in the S1-24 culms was 27% higher than in the wild type. The main function of cellulose is mechanical support, so the low mechanical strength in the S1-24 mutant was caused primarily by decreased cellulose content. The increase in hemicellulose in the S1-24 mutant may be the result of a compensation reaction. The mutated gene in the S1-24 mutant appeared to play an important role in cellulose biosynthesis and to affect cell wall morphogenesis.

### Genetic analysis and gene mapping

**Fig 5:**
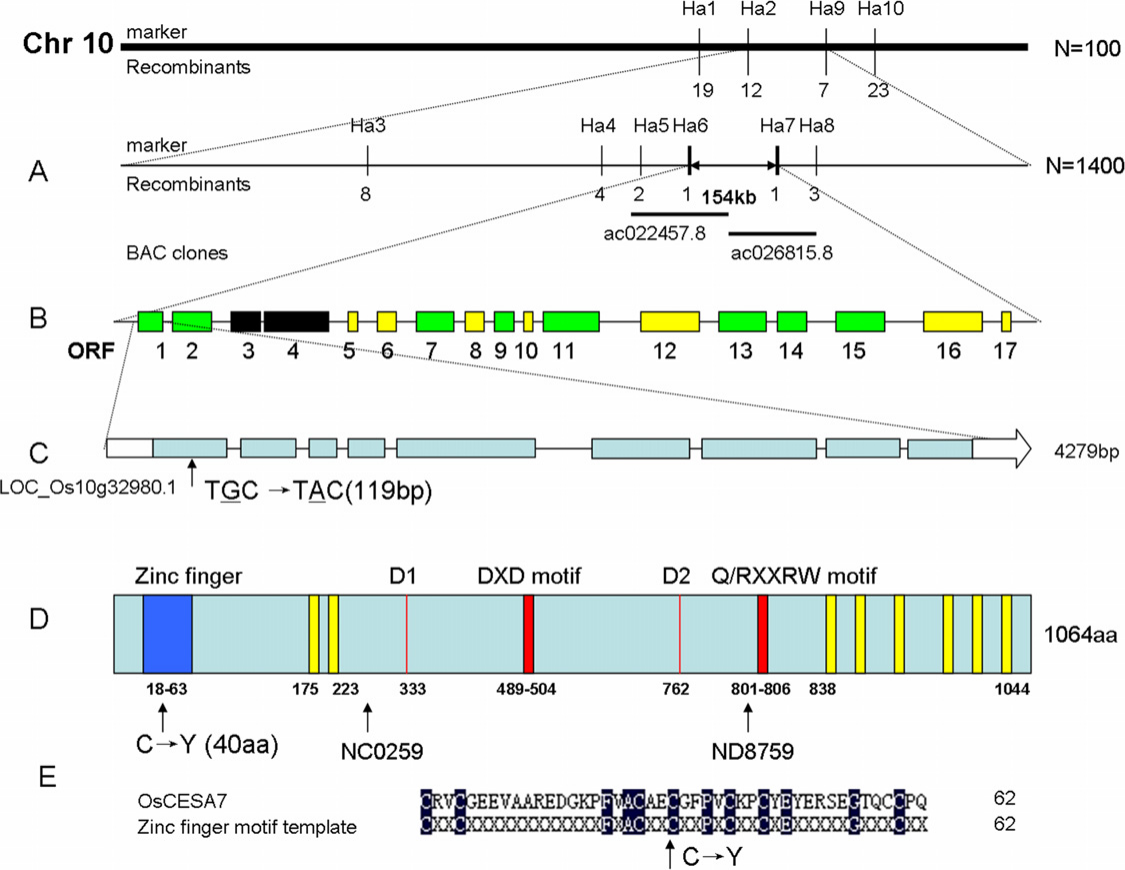
Map-based cloning of the gene responsible for the S1-24 phenotype. (A) The location of the gene locus was narrowed to an approximately 154-kb region on chromosome 10. Vertical lines represent the positions of molecular markers and the number of recombinants. (B) Seventeen predicted ORFs within the fine mapping region. Green, ORFs with known biochemical functions; Yellow, ORFs encoding expressed hypothetical proteins; Black, ORFs encoding transposons. (C) Genomic structure of *OsCESA7*. Boxes indicate exons. The mutation site is located in the first exon. (D) Protein structure of OsCESA7 including the RING-type zinc finger indicated in blue; two Asp (D) residues, the DXD, Q/RXXRW motifs indicated in red; and eight transmembrane domains indicated in yellow. Tos17 insertion sites in the NC0259 and ND8759 mutants allelic to the S1-24 mutant are indicated by arrows. (E) Alignment of zinc finger motif template and the corresponding OsCESA7 region. The site of the mutation C40 is highly conserved.

To determine the genetic basis of the S1-24 mutation and clone the mutated gene, an F_2_ population was generated by crossing S1-24 with Dular, a polymorphic *indica* variety. In the F_2_ population, normal and brittle culm phenotypes were exhibited by 4,934 and 1,550 individual plants, respectively, in accordance with a Mendelian segregation ratio of 3:1 (χ^2^ = 2.10 < χ^2^_0.05_ = 3.84), indicating that the brittle culm phenotype is controlled by a single recessive gene. A map-based cloning approach was used to isolate the causative gene. For primary mapping, 100 F_2_ mutant individuals were used to locate the target gene between two indel markers, Ha2 and Ha9, on the long arm of chromosome 10. Six new indel markers and 1,400 F_2_ individuals with the brittle culm phenotype were used for fine mapping. The gene was localized within a 154-kb region between indel markers Ha6 and Ha7 (Fig 5A, S1 Table). This region was covered by overlapping the BAC clones Ac022457.8 and Ac026815.8.

### A missense mutation in OsCESA7 in the S1-24 mutant

According to the MSU Rice Genome Annotation Release 7 (http://rice.plantbiology.msu.edu), 17 predicted open reading frames (ORFs) were present in the 154-kb interval (Fig 5B). In this region, eight ORFs with known biochemical functions, seven ORFs encoding expressed hypothetical proteins, and two transposons were predicted (Fig 5B, S2 Table). After analysis, ORF1 (LOC_Os10g32980.1) was selected. This gene encoded cellulose synthase A catalytic subunit 7 (*OsCESA7*), which is a key gene in cellulose biosynthesis, consistent with the brittle culm phenotype. Therefore, we sequenced the gene in the S1-24 mutant and compared the sequence to wild-type alleles of the *OsCESA7* gene. The *OsCESA7* gene is 4,279 bp in length and has nine exons and eight introns (Fig 5C). A single base pair substitution was found in the first exon that resulted in a codon change from TGC to TAC with a corresponding change in the encoded amino acid from cysteine to tyrosine at amino acid position 40 (Fig 5D).

The protein encoded by *OsCESA7* is 1,064 amino acids in length. A RING-type zinc finger is present in the N-terminal region. A large central domain contains two Asp (D) residues and the DXD and Q/RXXRW motifs that are critical for catalytic activity of the enzyme. In addition, the protein contains eight putative transmembrane domains (TMDs) with two located near the amino terminus and six clustered near the carboxyl terminus (Fig 5D) [25]. The missense mutation in S1-24 is located in the RING-type zinc finger domain and is strictly conserved in the zinc finger motif template (Fig 5E). This domain was predicted to mediate the interaction among different CESA subunits [26]. The mutation might affect the interaction between OsCESA7 and other OsCESA subunits.

### Complementation analysis

**Fig 6:**
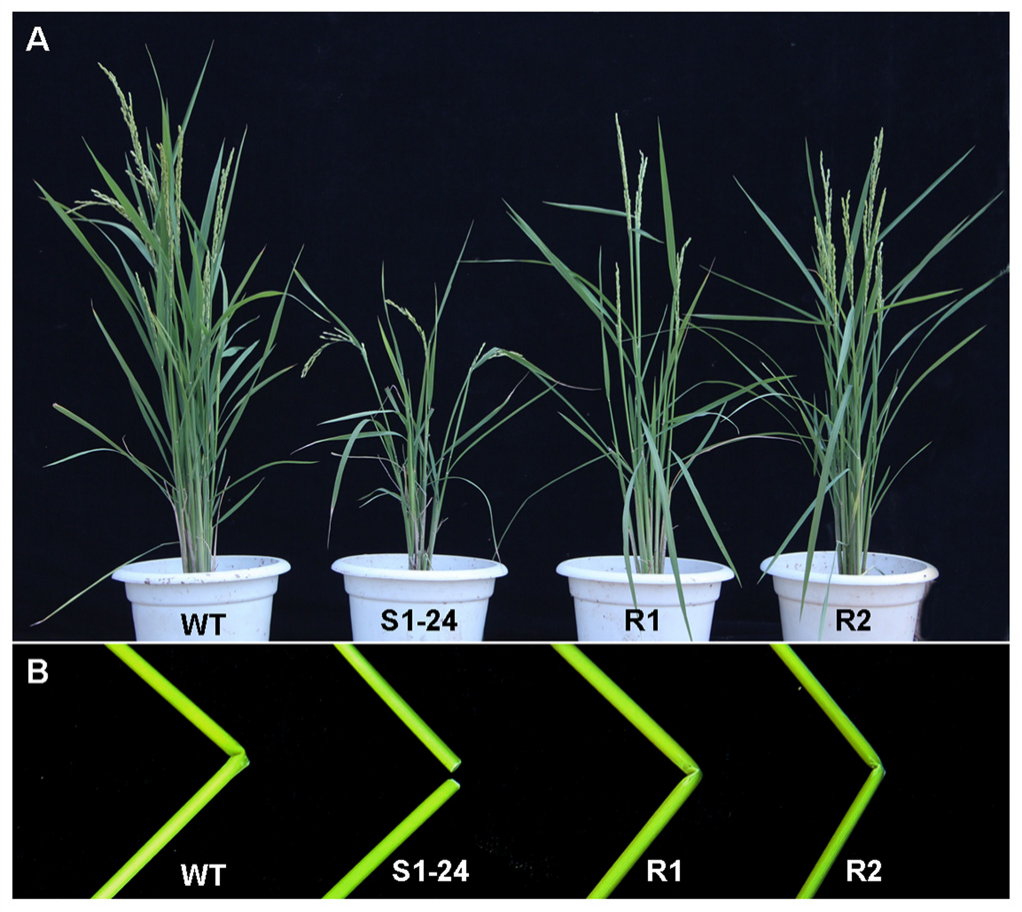
Phenotypes of genetic complementation in transgenic S1-24 mutant plants. (A) Gross morphologies of wild-type, S1-24, and R1 and R2 complementation lines at the mature stage. (B) Resistance to breakage in culms of the R1 and R2 complementation lines compared with S1-24 and wild-type culms.

The identity of *OsCESA7* was confirmed by genetic complementation analysis. A plasmid containing the entire *OsCESA7* coding region and promoter was introduced into the S1-24 mutant. Approximately 10 lines and 100 independent transgenic plants were obtained. Eight lines, including R1 and R2, showed restoration of the wild-type phenotype in the S1-24 mutant background. The R1 and R2 lines exhibited phenotypes similar to the wild type, the most obvious being the absence of brittle culms (Fig 6). Therefore, we conclude that the mutation in *OsCESA7* leads to the brittle culm phenotype.

### OsCESA7 expression pattern

**Fig 7:**
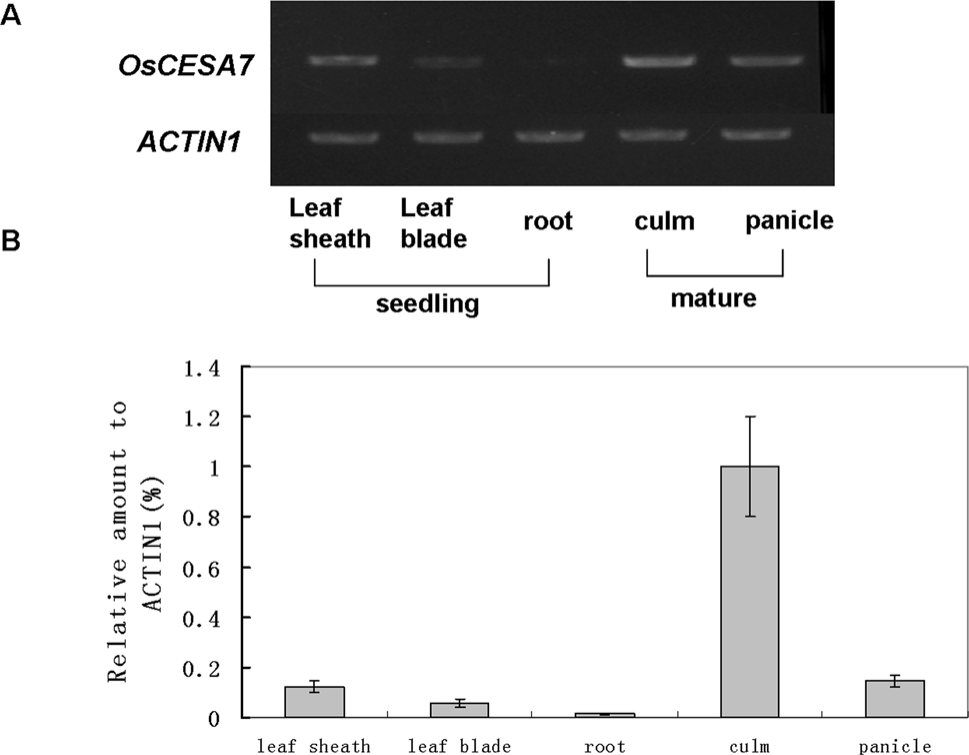
RT-PCR analysis of *OsCESA7* expression. (A) Semiquantitative RT-PCR analysis of *OsCESA9* expression. (B) Real-time RT-PCR analysis of *OsCESA7* expression. Total RNA was extracted from leaf sheaths, leaf blades, and roots at the seedling stage and from culms and panicles at the mature stage of wild-type plants. The rice *ACTIN1* gene was used as a control for equal loading. Values shown are averages of four replicates. Bars represent standard errors.

**Fig 8:**
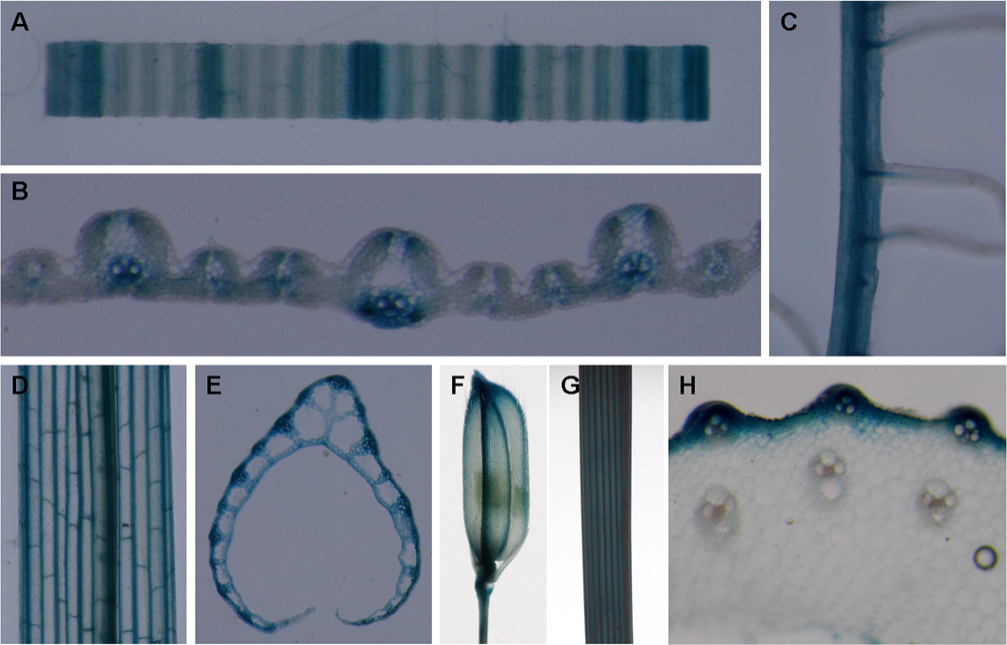
Expression pattern of OsCESA7 revealed by GUS-staining in OsCESA7promoter: GUS transgenic plants. (A) A segment of leaf blade. (B) Leaf blade cross section. (C) Magnified image of the root. (D) Leaf sheath. (E) Leaf sheath cross section. (F) Spikelet. (G) Stem. (H) Stem cross section. Signals were detected in vascular bundles, especially in sclerenchyma cells.

The expression levels of *OsCESA7* in various rice organs were examined by semiquantitative and quantitative RT-PCR. The *OsCESA7* gene was expressed predominantly in the culm of mature stage plants, consistent with the brittle culm phenotype in the S1-24 mutant. In addition, the *OsCESA7* gene was also highly expressed in panicles at the mature stage. In contrast, expression levels were relatively low in roots, leaf blades, and leaf sheaths at the seedling stage (Fig 7). Furthermore, detailed expression pattern of the *OsCESA7* gene was examined by histological staining of the transgenic plants harboring the *OsCESA7* promoter driven GUS reporter gene. In total, 5 transgenic lines and 48 independent transgenic plants were obtained, and GUS signals were found to be positive in 38 plants of 4 lines. As shown in Fig 8, GUS signals were observed primarily in mechanical tissues, such as vascular bundles in leaf blades (Fig 8A and 8B), leaf sheaths (Fig 8D and 8E), glumes (Fig 8F), roots (Fig 8C), and sclerenchyma cells in the outer layers of culms (Fig 8G and 8H). These observations indicate that OsCESA7 is expressed throughout the plant with high expression in mechanical tissues.

### Alignment and phylogenetic analysis

**Fig 9:**
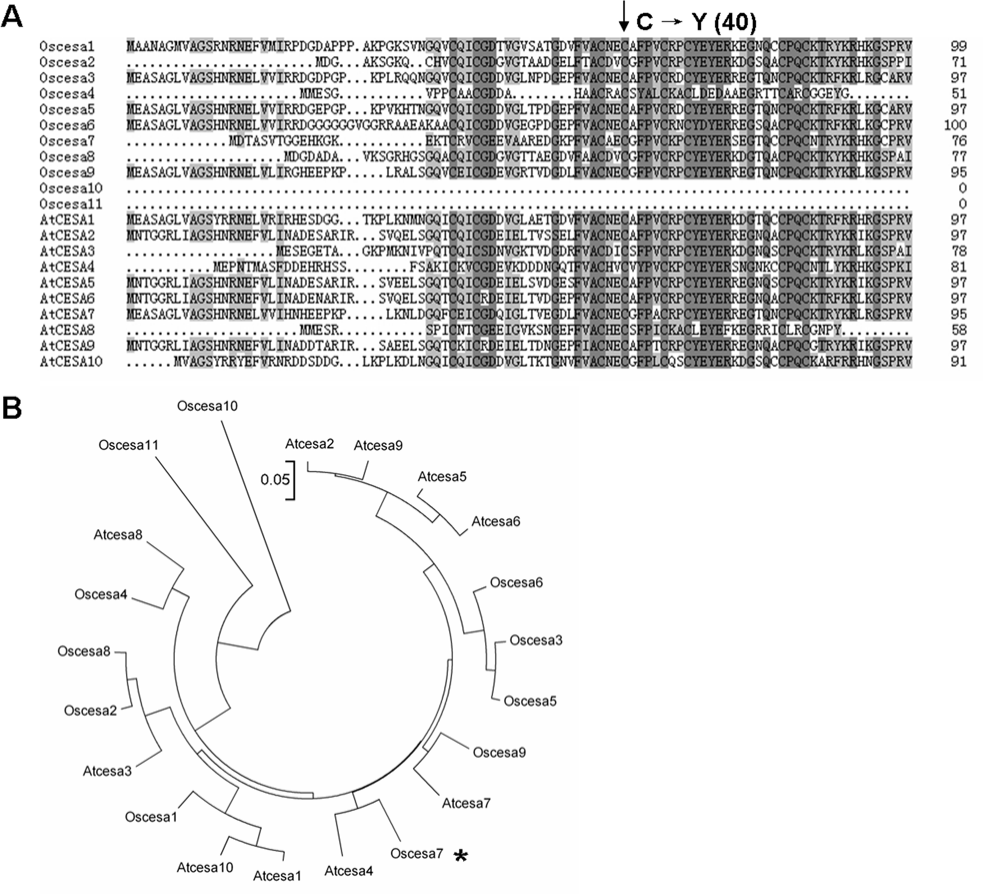
The site of the mutation in the S1-24 mutant and phylogenetic analysis of OsCESA7. (A) Multiple alignments of the N-terminal region of 11 members of the OsCESA family and 8 members of the AtCESA family. The mutated residue (cysteine 40) is highly conserved. (B) Phylogenetic analysis of CESAs. The scale bar is an indicator of genetic distance based on branch length.

Multiple alignments revealed that the mutation site (cysteine) at amino acid position 40 of OsCESA7 in the S1-24 mutant is highly conserved in 19 of 21 members of the *OsCESA* and *AtCESA* gene families (Fig 9A, S3 Table). This strong conservation indicates that this residue in the OsCESA zinc finger domain is important and necessary. We analyzed the evolutionary relationships among the CESA families in rice and *Arabidopsis*. As shown in Fig 9B, CESA members clustered into several clades. OsCESA4, OsCESA7, and OsCESA9, for which mutants have been identified and characterized [14–17], belonged to a monophyletic clade and was most closely related to AtCESA8, AtCESA4, and AtCESA7, respectively, while other CESAs were distributed in clades of three or four members. These results indicate that functions of some CESA members are redundant and that others are distinctive. Cellulose synthesis requires the cooperation of at least two to three different *CESA* gene family members [4, 6]. Diverse *CESA* genes would enable the formation of multiple subunit complexes essential for cellulose synthesis in cell wall biosynthesis.

## Discussion

The cell wall is a system of fibers that support cellular tissues, and both the structure and composition of the cell wall are important to its function. To examine the relationship between mechanical strength and the mechanism of cell wall biosynthesis, we isolated mutants with reduced mechanical strength. Biochemical and molecular characterization of these mutants revealed that formation of the brittle culm phenotype was caused by defects in cellulose synthesis. CESA is an absolutely necessary factor in the synthesis of cellulose. To date, many *CESA* genes have been identified. At least 11 *OsCESA* genes are present in rice, and brittle culm mutants resulting from the insertion of a *Tos17* retrotransposon into the *OsCESA4*, *OsCESA7*, and *OsCESA9* genes have been reported [14]. In addition, *bc7* and *bc11* are allelic mutants of *OsCESA4*, and *bc6* is a mutant of *OsCESA9*, but no corresponding studies have been performed for *OsCESA7*.

In this study, we isolated a brittle culm rice mutant, S1-24, from EMS-mutagenized *japonica* cultivar Nipponbare. The mechanical strength of the culm was significantly reduced in the mutant (Fig 1). Compared with wild type, the cellulose content was decreased by 48%, and thickening of the sclerenchyma cell wall was defective in the mutant (Figs 3 and 4). Map-based cloning identified the mutated gene as *OsCESA7*, which encodes cellulose synthase A catalytic subunit 7 (Fig 5). In addition to the brittle phenotype, S1-24 also exhibited reduced plant height and tiller number with withered leaf tips and a decreased rate of seed setting (Fig 2).

In rice, two allelic mutants (NC0259 and ND8759) of *OsCESA7* were reported, but phenotype severity varied depended on the mutation site [14]. Cellulose content was reduced by 76% and 74.5% in NC0259 and ND8759, respectively. Plant growth was also severely affected, with mutant plants showing dwarfism, small leaves, withered leaf tips, and thin culms. The endogenous retrotransposon *Tos17* was inserted into the fifth exon in NC0259 and the seventh exon in ND8759, causing loss function of the central motif required for catalytic activity of the enzyme. The S1-24 mutant reported here is a weaker allelic mutant of NC0259 and ND8759, which exhibits a 48% reduction in cellulose content relative to the wild type. Other phenotypes of S1-24 such as dwarf and withered leaf tips were also weaker. This phenotypic difference may be explained in that the missense mutation C40Y in S1-24 occurring in the RING-type zinc finger motif near the amino terminus may affect the interaction of OsCESA7 with other CESA subunits, but the central motif required for catalytic activity of the enzyme is preserved. Characterization of multiple allelic mutants that occur at different sites in *OsCESA7* revealed that different mutation sites lead to variable phenotypes and that distinct motifs or residues have specific functions in the maintenance of CESA catalytic activity.

Cellulose synthase proteins are components of CESA complexes (rosettes) and are thought to catalyze the chain elongation step in glucan polymerization [27]. The N-terminal region of each CESA protein contains a RING-type zinc finger, which is a cysteine-rich domain. RING fingers have been implicated in mediating the interactions among a wide variety of proteins, including protein–protein interactions between the CESAs [26, 28]. Meanwhile, the N-terminal domain is structurally multifarious in CESAs because of the observation that dimerization of other types of proteins occurs via zinc fingers. Until now, the interactions between different CESA subunits were shown by various studies. In cotton, the N-terminal domain of the GhCESA1 protein was reported to be able to interact with itself and GhCESA2 in a two-hybrid system and pull-down experiments [26]. In *Arabidopsis*, AtCESA4, AtCESA7, and AtCESA8 proteins were able to be co-expressed and interacted with each other [13]. Coprecipitation of IRX1 with IRX3 indicated that IRX1 and IRX3 are part of the same complex [3]. The association of IRX1 and IRX3 decreased in the absence of IRX5, showing that IRX5, IRX3, and IRX1 are all essential components of the cellulose synthesizing complex, and that the presence of all three subunits is required for the correct assembly of this complex [29]. As OsCESA4, OsCESA7, and OsCESA9 were high homologous with IRX1, IRX5, and IRX3, similar intractions may also exist among OsCESAs. Besides, the functions of the CESA N-terminus were revealed to be varied. As reported, the zinc finger-like domain of AtCESA2 could homodimerize, possibly contributing to rosette assemblies of cellulose synthase A within plasma membranes [30]; A point mutation in the N-terminus of OsCESA9 could result in brittle culm phenotypes and reduced cadmium accumulation in rice [31]. However, no *OsCESA7* mutants occurring at the RING-type zinc finger of CESAs have been reported in either *Arabidopsis* or rice so far [28, 31, 32]. Therefore, S1-24 is considered to be the first mutant in the zinc finger of OsCESA7, and this mutant was able to influence both cellulose biosynthesis and plant growth in rice. The S1-24 mutant will be a useful genetic material for studying the interactions among different CESA subunits within the cellulose synthesis complex.

## Supporting Information

S1 Table The primer sequence used in genetic mapping. (DOC)

S2 Table Predicted ORFs in the fine mapping region. (DOC)

S3 Table Locus name of 11 *OsCESA* and *AtCESA* genes. (DOC)
