# Supplementary material for: A Missense Mutation in the Zinc Finger Domain of OsCESA7 Deleteriously Affects Cellulose Biosynthesis and Plant Growth in Rice
Source: PLoS One. 2016 Apr 19;11(4):e0153993. doi: 10.1371/journal.pone.0153993 (PMC4836682; doi:10.1371/journal.pone.0153993)
Supplement: S1 Table — (DOC) [file pone.0153993.s001.doc]

Table S1. The primer sequence used in genetic mapping

| marker | Forward primer | | Reverse primer | Length (bp) |
| --- | --- | --- | --- | --- |
| | Ha1 | | --- | | Ha2 | | Ha3 | | Ha4 | | Ha5 | | Ha6 | | Ha7 | | Ha8 | | Ha9 | | Ha10 | | | tcggctacacaaagtcgagc  tcaaataagacgaatggtca  tctctgccattcaccattga  tctagaatgtcactatggcc  ttcatcagagcatggcgtgt  tgacatggccacgctgacat  atggtcaaacgttaaacgtg  aagatgaggcaacccaagca  atgtccatagatacgtgcca  tgcagatgactgaaagccac | acgtggtactggtcaacgat  tgctataaccacccaaacct  tgaatctgagaacttcagct  aggatgtcgagctcagatct  cgtgctacctcaatggagaa  ccctcaacttgtcatcgagt  ctggtttctgtgtccatctt  acacctccttacctagacgt  atgcatgtcggtgcatgctg  gtcactgaactcacatgtca | 237  214  182  201  204  173  205  207  213  191 |
