# Supplementary material for: A Missense Mutation in the Zinc Finger Domain of OsCESA7 Deleteriously Affects Cellulose Biosynthesis and Plant Growth in Rice
Source: PLoS One. 2016 Apr 19;11(4):e0153993. doi: 10.1371/journal.pone.0153993 (PMC4836682; doi:10.1371/journal.pone.0153993)
Supplement: S2 Table — (DOC) [file pone.0153993.s002.doc]

Table S2. Predicted ORFs in the fine mapping region.

| ORF | Locus ID | Function annotation |
| --- | --- | --- |
| 1 | LOC_Os10g32980 | CESA7 - cellulose synthase |
| 2 | LOC_Os10g32990 | receptor-like protein kinase 2 precursor |
| 3 | LOC_Os10g33000 | retrotransposon protein |
| 4 | LOC_Os10g33010 | retrotransposon protein |
| 5 | LOC_Os10g33020 | expressed protein |
| 6 | LOC_Os10g33030 | expressed protein |
| 7 | LOC_Os10g33040 | receptor-like protein kinase precursor |
| 8 | LOC_Os10g33050 | expressed protein |
| 9 | LOC_Os10g33060 | hcr2-5D |
| 10 | LOC_Os10g33070 | expressed protein |
| 11 | LOC_Os10g33080 | leucine-rich repeat receptor protein kinase EXS precursor |
| 12 | LOC_Os10g33104 | expressed protein |
| 13 | LOC_Os10g33130 | leucine-rich repeat receptor protein kinase EXS precursor |
| 14 | LOC_Os10g33140 | hcrVf2 protein |
| 15 | LOC_Os10g33170 | POT domain containing peptide transporter |
| 16 | LOC_Os10g33190 | expressed protein |
| 17 | LOC_Os10g33200 | expressed protein |
