# Supplementary material for: A Missense Mutation in the Zinc Finger Domain of OsCESA7 Deleteriously Affects Cellulose Biosynthesis and Plant Growth in Rice
Source: PLoS One. 2016 Apr 19;11(4):e0153993. doi: 10.1371/journal.pone.0153993 (PMC4836682; doi:10.1371/journal.pone.0153993)
Supplement: S3 Table — (DOC) [file pone.0153993.s003.doc]

Table S3: Locus name of 11 *OsCESA* and *AtCESA* genes

| *OsCESA* number | MSU Locus ID | *AtCESA* number | TAIR Locus ID |
| --- | --- | --- | --- |
| 1 | LOC_Os05g08370 | 1 | AT4G32410.1 |
| 2 | LOC_Os03g59340 | 2 | AT4G39350.1 |
| 3 | LOC_Os07g24190 | 3 | AT5G05170.1 |
| 4 | LOC_Os01g54620 | 4 | AT5G44030.1 |
| 5 | LOC_Os03g62090 | 5 | AT5G09870.1 |
| 6 | LOC_Os07g14850 | 6 | AT5G64740.1 |
| 7 | LOC_Os10g32980 | 7 | AT5G17420.1 |
| 8 | LOC_Os07g10770 | 8 | AT4G18780.1 |
| 9 | LOC_Os09g25490 | 9 | AT2G21770.1 |
| 10 | LOC_Os12g29300 | 10 | AT2G25540.1 |
| 11 | LOC_Os06g39970 |  |  |
